# A Systematic Review of the Evaluation of Interventions to Tackle Children’s Food Insecurity

**DOI:** 10.1007/s13668-019-0258-1

**Published:** 2019-02-14

**Authors:** Clare E. Holley, Carolynne Mason

**Affiliations:** School of Sport, Exercise and Health Sciences, Loughborough University, Epinal Way, Loughborough, Leicestershire LE11 3TU UK

**Keywords:** Child, Food insecurity, Hunger, Intervention, Evaluation

## Abstract

**Purpose of Review:**

To synthesise the research which has sought to evaluate interventions aiming to tackle children’s food insecurity and the contribution of this research to evidencing the effectiveness of such interventions.

**Recent Findings:**

The majority of studies in this review were quantitative, non-randomised studies, including cohort studies. Issues with non-complete outcome data, measurement of duration of participation in interventions, and accounting for confounds are common in these evaluation studies. Despite the limitations of the current evidence base, the papers that were reviewed provide evidence for multiple positive outcomes for children participating in attended and subsidy interventions, inter alia, reductions in food insecurity, poor health and obesity. However, current evaluations may overlook key areas of impact of these interventions on the lives and outcomes of participating children.

**Summary:**

This review suggests that the current evidence base which evaluates food insecurity interventions for children is both mixed and limited in scope and quality. In particular, the outcomes measured are narrow, and many papers have methodological limitations. With this in mind, a systems-based approach to both implementation and evaluation of food poverty interventions is recommended.

## Introduction

Food insecurity is defined as “limited or uncertain availability of nutritionally adequate and safe foods or limited or uncertain ability to acquire acceptable foods in socially acceptable ways (e.g. without resorting to emergency food supplies, scavenging, stealing or other coping strategies)” [1]. Despite experiencing relative wealth as nations, food insecurity is an increasingly common phenomenon in some developed countries. Recent statistics indicate that in 2016, 12.3% of households and 8% of children in America experienced food insecurity [2]. Similarly, 19% of UK children under 15 live with a respondent who is moderately or severely food insecure and 10% live with a respondent who is severely food insecure [3]. Whilst there is no current data on levels of children’s food insecurity in the UK, eligibility for free school meals (which is based upon low household income) can be used as a proxy measure. In 2018, 13.6% of UK school children were eligible for free school meals [4], suggesting that food insecurity may also be a significant issue among UK children.

It is well-evidenced that food insecurity results in a restricted and less nutritionally adequate diet [5]. This has health implications, as children who experience food insecurity are likely to have poorer general health, approximately twice as likely to have asthma and almost three times as likely to have iron deficiency anaemia than food-secure children [6–8]. Children who experience food insufficiency are also significantly more likely to exhibit behavioural problems, have difficulty getting on with other children [9] and experience anxiety and depression [9, 10]. Moreover, in 2015, only 33.1% of UK school children eligible for free school meals achieved the key attainment indicator at the end of secondary school, compared to 60.9% of more food-secure school children [11].

The evidence base described provides a compelling case for interventions that seek to tackle children’s food insecurity, in order to minimise the health and social disparities between children who experience food insecurity and those who do not.^1^ These interventions take multiple formats; from attended interventions (e.g. school food assistance and holiday clubs) to providing disadvantaged families with subsidies (e.g. the Supplemental Nutrition Assistance Program or SNAP). However, there is currently a lack of synthesis of the evidence base which examines the effectiveness of these interventions, particularly regarding the ways in which effectiveness is evaluated.

To our knowledge, two systematic reviews of interventions to tackle children’s food insecurity have been published to date. One is a rapid review recently published which only included interventions in the form of charitable breakfast clubs and holiday hunger projects in the UK [12••]. The other is a recent rapid review funded by the NIHR which explores the nature, extent and consequences of food insecurity among children [13]. However, this review focused on quantitative outcomes of interventions where the food insecurity status of the sample has been measured and, therefore, only includes a very targeted and limited number of studies. With this in mind, the current review sought to produce a systematic review of the literature on interventions from developed countries which seek to tackle children’s food insecurity, to gain a clear picture of the following: (1) the ways in which food insecurity interventions are evaluated; and the quality of this evaluation and (2) the evidence base of the impact of these interventions, in terms of positive outcomes for the targeted children.

## Methods

### Search Strategy

Online searches were conducted using three databases to ensure that the full breadth of relevant publications was identified. These databases were PsycINFO, Medline and Scopus. Key terms relating to food insecurity interventions were used to identify a pool of potentially relevant papers for this review. These key words were the following: child* adolescent* “young people” “youth” “intervention” “holiday club” combined using the Boolean operator AND with any of the following words: “food insecurity” OR “food poverty” OR “food insufficiency” OR “holiday hunger”. Relevant articles were detected up until July 2018.

### Inclusion and Exclusion Criteria

To be included in this review, papers were required to evaluate interventions that seek to tackle food insecurity and to measure the outcomes specifically for children. Studies were also required to take place in a developed country as defined by the United Nations [14] and to be published in a peer-reviewed journal. Papers were excluded if they only measured children’s uptake of an intervention (e.g. a process evaluation) or if the outcome measures referred to households or families rather than children. They were also excluded if they were not published in English.

### Identification of Relevant Papers

**Fig. 1:**
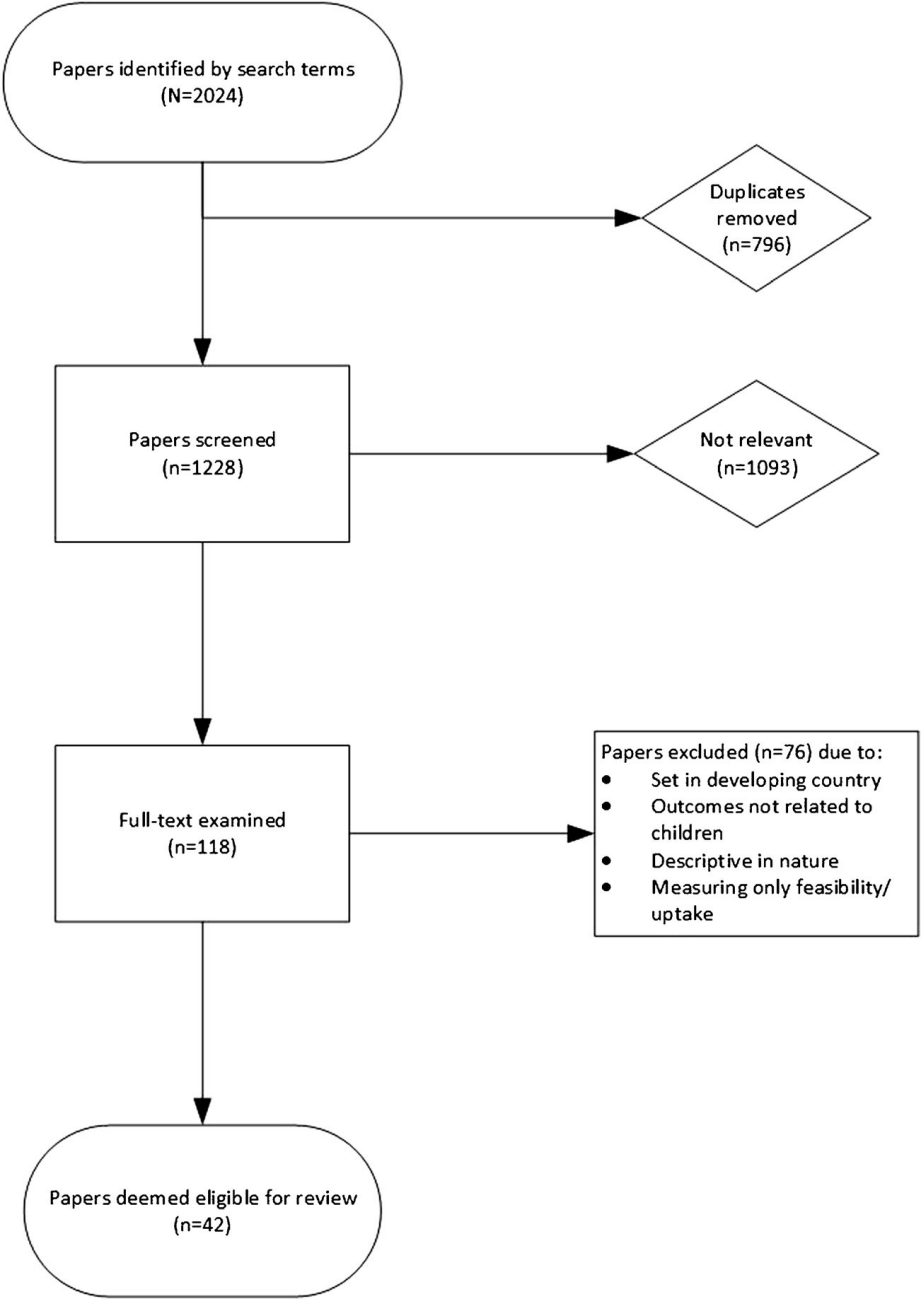
Flow diagram of the identification process for papers included in this systematic review

The process for identification of relevant papers can be seen in Fig. 1. The first author screened the titles of all the search results to identify potentially relevant papers. When a title was deemed relevant (or when relevance was ambiguous), the abstract was screened for eligibility to assess whether the paper met the inclusion and exclusion criteria. The full text was then read for all papers which either (a) met these criteria or (b) did not contain sufficient detail in the abstract to assess eligibility.

### Data Extraction

Data was extracted by the first author from 42 papers that met the eligibility criteria for this review. Data extracted was standardised across studies using a form specifically developed to meet the aims of this review. Extracted data included author(s), date and place of publication, country of study, study aim(s), type of intervention, target population of intervention, method of evaluation and the findings pertaining to child outcomes of the intervention(s). A summary of this data can be seen in Tables 1 and 2 for attended (in person) and subsidy-based interventions respectively.^2^

**Table 1:** Summary of extracted data from papers that evaluate attended interventions which seek to tackle children’s food insecurity

Authors	Year	Country	Intervention	Target	Design (data source if secondary data)	Subjects	Findings
Bruce et al.	2017	US	Summer library meal programme	Children, parents and other adults	Mixed methods	67 adults (89% parents)	Parents value library lunch scheme in the summer holidays as a method of enrichment for children, allowing socialisation
Carney et al.	2012	US	Community gardening programme	Migrant seasonal farmworker families	Mixed methods	38 families	Frequency of children’s vegetable intake of “several time a day” increased from 24.0 to 64.0%. No significant change in the reported number of meals children skipped due to lack of money
Corcoran et al.	2016	US	Breakfast in the Classroom	School children	Experimental	Unknown	No evidence of gains in academic performance or of feared increases in obesity
Dave et al.	2018	US	Kid’s Café Program	Children	Cluster Randomised Control Trial (RCT)	120 9–12-year-olds	At post-test, children from both groups had significantly lower vegetable consumption, intervention group children had significantly higher sodium scores. No significant difference in BMI after intervention or between groups
Defeyter et al.	2015	UK	Holiday breakfast clubs	Children and adults	Qualitative: semi-structured interviews	17 children 18 adult attendees 15 staff	Staff and attendees report nutritional, social and financial benefits. Attendees consume greater variety of breakfast foods and attendance lessens social isolation.
Dunifon et al.	2002	US	National School Lunch Program	Children	Cross-sectional (Child Development Supplement—Panel Study of Income Dynamics: CDS-PSID)	1618 children aged 6–12 years	Participating in NSLP associated with increased odds of externalising behaviour, 82% increased odds of a health limitation and lower maths test scores. Associations disappear when comparing the outcomes of siblings, one of whom does not participate in NSLP.
Fletcher et al.	2017	US	School Breakfast Program	Children	Cross-sectional (National Health and Nutrition Examination Survey: NHANES)	2734 children aged 6–16	Access to SBP reduces the likelihood of children’s very low food security by 2% (baseline rate of 2%, so a 100% reduction).
Graham et al.	2016	UK	Holiday clubs	Children	Qualitative: semi-structured interviews	14 staff members	Staff perceive multiple benefits to children attending holiday clubs including food (trying new foods, more nutritious foods, attenuating hunger), activities social opportunities, informal learning
Gundersen et al.	2012	US	National School Lunch Program (NSLP)	School children	Cross-sectional (NHANES)	3796 6–17-year-olds	Free lunch programme reduces incidence of poor health by between 33 and 67%, obesity by at least 21%, and food poverty by at least 6%.
Harvey-Golding et al.	2016	UK	Universal Free School Breakfast (UFSB)	School children	Qualitative: semi-structured interviews	19 senior stakeholders from local authorities	Stakeholders perceive UFSB as alleviating hunger, improving health outcomes, improving finances, and having potential for educational, social and behavioural benefits. Stakeholders also reported concerns about potential increases in obesity.
Jones et al.	2003	US	School lunch programme, school breakfast programme and FSP	Children (and families in the FSP)	Cross-sectional (CDS-PSID)	772 5–12-year-old children	Food-insecure girls who participated in all 3 programmes had 68% reduced odds of being at risk of overweight. Participation had no association with risk of overweight for girls in food-secure households or any boys.
Kastorini et al.	2016	Greece	“DIATROFI” programme: provides all students of participating schools with daily free meals and health education for participants and families	School children (and families)	Experimental-no control	162 schools in low SES areas (3941 pre- and post-questionnaires completed)	Frequency of consumption of all foods promoted by the intervention significantly increased (milk/yoghurt: 16.7%; fruits: 17.5%; vegetables: 16.9%; whole grain products: 85.7%), and diet quality increased among participating adolescent girls.
Khan et al.	2011	US	School breakfast and lunch provision	School children	Cross-sectional	373 students aged 11 to 14	School breakfast provision attenuates group differences in breakfast consumption among food-insecure children.
Kohn et al.	2014	US	SNAP WIC school meals	Children/mothers	Cross-sectional (NHANES)	1321 children aged 4 to 17 from low income families	Food assistance programme participation is associated with increased BMI-Z and waist circumference among food-secure children, but not among food-insecure children.
Munday et al.	2017	New Zealand	Nutrition education	Children	Experimental-baseline vs post-intervention with 6-month follow-up	17 kindergarten children (mean 4 years and 2 months)	No significant changes in nutrient intake, but a significant decrease in consumption of ultra-processed snack foods. Also increases of fruit and vegetable consumption at 6 months (not tested for statistical significance).
Nalty et al.	2013	US	SNAP, NSLP	Children/mothers	Longitudinal questionnaire study	40 mother–child dyads	School-based nutrition programmes (WIC, NSLP, SBP) reduced the odds of child food insecurity by 74%
Nguyen et al.	2017	US	SNAP, NSLP	Low-income households	Questionnaire study	4719 children and adolescents	Among SNAP participants and non-participants, there was no significant relationship between household food security and BMI.
Racine et al.	2013	US	Food and Fun	Mothers and children	Experimental-no control	7 mothers and 13 children	Significant increase in children’s fruit and vegetable consumption (increase of 2.2 portions per day) and physical activity (additional 2 days reported as active for more than 1 hour)
Rivera et al.	2016	US	SNAP-Education	Low-income households	RCT	208 households	No significant change in child food security status in comparison to control group. Authors suggest that children are protected from worst effects of household food insecurity, and that children’s food insecurity was low
Rodriguez et al.	2013	US	Nutrition education	Homeless children	Experimental	162 children	Some indication of an increase in children’s willingness to try different foods, liking new foods, and new intentions to change health behaviours among attending children
Roustit et al.	2010	Canada	School food programme	Children	Cross-sectional (Social and Health Survey of Quebec Children and Adolescents)	2346 13- or 16-year-olds	Household food insecurity associated with scholastic difficulties. No association for children attending schools with food assistance. Attendees also had reduced risk of school activity limitation, repeating a year, below-average grades and poor academic performance all decreased.
Sharkey et al.	2013	US	NSLP, SNAP	Low-income households	Cross-sectional (colonia–household and community food resource assessment)	470 adults in Texas border region with at least one child in household	NSLP participation associated with increased odds of child hunger whilst SNAP participation associated with decreased odds for child hunger
Sharma et al.	2017	USA	Brighter Bites: aims to tackle food waste and improve access to fresh produce and nutrition education among underserved children and their families.	Underserved children and their families	Cross-sectional	2013–2014: 1224 families enrolled; 2014–2015: 2625 families enrolled; 2015–2016: 8216 families enrolled (unclear *n* for evaluation of data)	83% of parents reported availability of produce improved child intake of F&V, and > 70% reported nutrition education to be effective.
Shemilt et al.	2004	England	School Breakfast Club	Children	RCT	5525 children	Children attending schools with breakfast club provision had significantly better concentration post-intervention than controls. Significantly fewer intervention children reported skipping classes. Significantly more children attending breakfast club reported eating fruit for breakfast than controls. A significantly larger proportion of primary-school children who had attended breakfast club had conduct issues.

**Table 2:** Summary of extracted data from papers that evaluate subsidy interventions which seek to tackle children’s food insecurity

Authors	Year	Country	Intervention	Target	Design (data source if secondary data)	Sample	Findings
Beck et al.	2014	US	Keeping Infants Nourished and Developing (KIND)	Food-insecure families with infants.	Experimental	5071 infants	Completion of preventative care services and a full set of well-infant visits by 14 months more common among KIND users. No significant differences in weight-for-length.
Beharie et al.	2017	USA	Supplemental Nutrition Assistance Program (SNAP)	Low-income households	Cross-sectional (National Survey of Children’s Health)	25,899 children/households	SNAP participation moderates the association between material deprivation and grade retention.
Black et al.	2004	US	Women, Infants and Children (WIC) program	Mothers (pre- and post-partum), infants and children	Cross-sectional (The Children’s Sentinel Nutritional Assessment Program)	5923 infants	Length-for-age and height-for-age among WIC claimers in line with national average. WIC entitled but not claiming due to access issues were underweight and short and had 1.9 times odds of fair/poor health as opposed to good/excellent compared to those receiving WIC.
Black et al.	2012	US	WIC	Women and children	Cross-sectional (Children’s HealthWatch)	26,950 caregivers and children younger than 36 months	WIC participation attenuates child health risks associated with stressors, e.g. fair/poor health, well-child status and overweight.
Burgstahler et al.	2012	US	SNAP	Low-income households	Cross-sectional (survey of household finances and childhood obesity)	360 children aged 2–18	Participation in SNAP has a significant negative effect on weight status, depth and severity of overweight, with and without controlling for financial stress.
Collins et al.	2017	US	Summer Electronic Bank Transfer for Children (SEBTC)-SNAP extension	Low-income households	Randomised controlled trial (RCT)	9124 households	Very low food security among children reduced by 34% in SEBTC group. Moderate improvements in children’s fruit, vegetable and dairy consumption.
Cook et al.	2006	US	Food Stamp Program	Low-income families	Cross-sectional (The Children’s Sentinel Nutrition Assessment Program)	17,130 children	Participation in FSP reduced the odds of fair/poor health (as opposed to excellent/good health) by 24 and 42% in children in food-insecure households and food-insecure children in food-insecure households, respectively.
Frongillo et al.	2006	US	Food Stamp Program (FSP)	Low-income families	Longitudinal (early childhood longitudinal study—kindergarten cohort)	10,600 kindergarten children	Children who started participating in FSP had a 3-point greater improvement in both reading and mathematics scores than children who stopped FSP participation. When split by gender, only significant for females.
Gibson	2004	US	FSP	Low-income families	Longitudinal (National Longitudinal Survey of Youth 1979: NLSY79)	7843 children	Participation during all of the previous 5 years was associated with a 42.8% increase for young girls and a 28.8% decrease for young boys in the predicted probability of overweight. Long-term FSP participation was not significantly related to overweight in older children
Gundersen et al.	2017	US	SNAP	Low-income households	Longitudinal (survey of income and programme participation)	5653 households with children	SNAP decreases the percentage of children not eating enough by at least 5.1%, or 41%, relative to the baseline that SNAP did not exist.
Hanson et al.	2017	US	Cost-offset agriculture	Low-income households	Longitudinal experimental study: no control, national averages used as comparison	41 low-income households	Participating children had higher fruit and vegetable intake than the US averages and met daily recommendations for vegetable intake more often (82.9% met recommendations). Total fruit and vegetable intake did not differ between summer and winter.
Jones et al.	2003	US	School Lunch Program, School Breakfast Program and FSP	Children (and families in the FSP)	Cross-sectional (CDS-PSID)	772 5–12-year-old children	Food-insecure girls who participated in all 3 programmes had 68% reduced odds of being at risk of overweight. Participation had no association with risk of overweight for girls in food-secure households or any boys.
Kohn et al.	2014	US	SNAP, WIC, School meals'	Children/mothers	Cross-sectional (NHANES)	1321 children aged 4 to 17 from low-income families	Food assistance programme participation is associated with increased BMI-Z and waist circumference among food-secure children, but not among food-insecure children.
Korenman et al.	2013	USA	Child and Adult Care Food Program (CACFP)	Children	Cross-sectional (early childhood longitudinal study-birth cohort)	4250 4-year-olds	Among low-income children, CACFP participation moderately increases probability of RDA consumption of milk (6–7%) and vegetables (9–11%), and may also reduce the prevalence of overweight and underweight (effect of 0.5% reduced odds among low-income, non-head start children).
Klerman et al.	2017	US	SEBTC-SNAP	Households with school-age children who, in the prior school year, were certified for FRP school meals by the school food authorities	RCT	48,449 households	Weekly electronic benefit transfer reduced children’s very low food security by 1/3 and children’s food insecurity by 1/5. $60 benefit had a significantly larger effect on children’s food insecurity than $30, with the exception of children’s very low food security, for which $30 transfer accounted for 2.4 of the 3% reduction.
Kreider et al.	2012	US	SNAP	Low-income households	Cross-sectional: NHANES	4418 income-eligible children	Under the assumptions that individuals may participate in SNAP due to expecting to be food-insecure and that poor health weakly decreases with family resources, estimates indicate that the programme has reduced the prevalence of food insecurity by at least 28% and poor health by at least 54%.
Kreider et al.	2016	US	WIC	Women and children	Cohort study: NHANES	4614 children	WIC reduces the prevalence of child food insecurity by at least 3.6 percentage points (20%).
Loibl et al.	2017	US	The Individual Development Account (IDA) programme	Low-income families/individuals	Longitudinal experimental study	639 participants in the IDA scheme	No significant differences in levels of food insecurity based on programme status.
Mabli et al.	2014	US	SNAP	Low-income households	Experimental: new users vs current users	2717 households	SNAP participation associated with ~ 1/3 decrease in odds of child food insecurity and a decrease in the odds of children experiencing severe food insecurity.
Nalty et al.	2013	US	SNAP, NSLP	Children/mothers	Longitudinal questionnaire study	40 mother-child dyads	School-based nutrition programmes (WIC, NSLP, SBP) reduced the odds of child food insecurity by 74%.
Nguyen et al.	2017	US	SNAP and NSLP	Low-income households	Cross-sectional	4719 children and adolescents	Among SNAP participants and non-participants, there was no significant relationship between household food security and BMI. Among NSLP participants, non-significant trend towards increasing BMI percentile with decreasing household food security in those reporting less than 3 lunches per week
Sharkey et al.	2013	US	NSLP SNAP	Low-income households	Cross-sectional (colonia–household and community food resource assessment)	470 adults in Texas border region with at least one child in household	NSLP participation associated with increased odds of child hunger whilst SNAP participation associated with decreased odds for child hunger
Zhang et al.	2017	US	SNAP	Low-income households	Cross-sectional (Current Population Survey-Food Security Supplement-CPS-FSS)	1826 families	SNAP participation increases the probabilities of children’s low food security by 6.1% and very low food security by 2.7%.

### Quality Assessment

The first author performed an assessment of the quality of the final papers included in this review using the latest version of the Mixed Methods Appraisal Tool (MMAT) [15]. This tool provides five questions to assess the quality of studies with qualitative, quantitative or mixed methodologies. In line with the creating authors’ guidance, scores for individual papers were not calculated. Instead, analysis was undertaken on the quality of the included papers collectively.

## Results

The 42 papers included in this review were published between 2002 and 2018 and reported on studies conducted in the USA (*n* = 34), UK (*n* = 4), Australia (*n* = 1), Canada (*n* = 1), Greece (*n* = 1) and New Zealand (*n* = 1). The interventions took multiple formats that can be categorised into two groups, attended (in-person) interventions and subsidy interventions. First, the ways in which these papers evaluate food insecurity interventions are explored, as well as the quality of these papers. Next, the evidence base, as described in these papers, of the outcomes of these interventions for participating children is discussed.

### The Kinds of Evaluation of Food Insecurity Interventions Taking Place

Interventions captured in this review adopt different approaches that aim to tackle separate aspects of food insecurity in order to achieve particular positive outcomes for children, and this, therefore, informs the kind of evaluation which takes place. For example, school-based interventions that typically aim to tackle children not accessing an adequate breakfast or lunch and then examine the impact on children in the classroom (e.g. behaviour, educational achievement). Interventions that attempt to increase consumption of fruit and vegetables measure effectiveness in terms of achieving this aim, whilst ignoring any potential wider impacts that may impact positively (or negatively) on children’s outcomes.

The research methods used in the papers included in this review are listed in Tables 1 and 2. There is a paucity of large-scale RCTs to investigate the effectiveness of interventions for tackling children’s food insecurity, and for ethical reasons (such as withholding an intervention from individuals who it is believed would benefit), this would be difficult to overcome. Several studies captured in this review utilise cohort data to overcome these ethical issues. Whilst this allows comparison between participants and other individuals of a similar demographic, there are other methodological limitations of this approach. For example, as described in Dunifon and Kowaleski-Jones’ evaluation of the Nation School Lunch Program (NSLP), differences in outcomes between eligible children who do and do not participate in interventions may well be driven by unmeasured factors and selection bias [16]. To attenuate the issue of selection bias, as well as issues of identification (where it is not always possible to assess participation), the cohort studies included in this review, particularly those which explore relationships between subsidy intervention participation and children’s food insecurity and obesity, have used a range of statistical methods [16–19]. However, there is a lack of consensus as to which of these statistical methods is most appropriate for overcoming issues of bias within data, and this makes it difficult to assess the robustness of findings reported in these papers.

Several of the studies detected by this review are experimental studies that provide valuable quantitative results on the outcomes of the interventions seeking to tackle children’s food insecurity. However, these are often small-scale, with only parent-report/self-report measures of outcomes, such as dietary intake [20–23].

When considering the quantitative articles in this review as a whole, it is apparent that there is a lack of longitudinal analyses which can better unpack the causality of relationships between intervention participation and outcomes for children. The studies presented provide the beginning of an understanding of the possible benefits for children participating in food insecurity interventions. However, large-scale longitudinal studies which allow assessment of long-term outcomes and remove the potential confounds associated with group comparisons are strongly needed.

Qualitative studies have been conducted to gain service-user perspectives on food insecurity interventions and the positive benefits for children [24, 25, 26•, 27]. Whilst these studies play an important role in evaluating these interventions, those captured in this review have predominantly utilised parents and stakeholders as participants, whereas children’s voice is only included in a small proportion of these studies. In light of this, future research should seek to ensure that the voices of participating children and young people are included in evaluation work, as those best placed to report their own experiences [28].

### Quality Assessment

Utilising the MMAT (2018) revealed that there was variation between the attended and subsidy interventions in the quality of the studies that were undertaken. For each of the groups, 24 papers were reviewed in total. It is important to note that as the methods varied for the studies, the quality criteria also varied across the studies. However, examining the number of papers that met all five criteria associated with the particular study design is useful as a way of exploring the overall quality of the evidence base. For the subsidy programmes, 95% of the papers met at least three of the quality criteria whilst a smaller number met the third (58%) and fourth (50%) quality criteria. For the attended programmes, there was less divergence and more consistency across studies in meeting the quality criteria (criteria 1 = 75%, criteria 2 = 71%, criteria 3 = 83%, criteria 4 = 63%, criteria 5 = 79%.)

The majority of the studies (*n* = 32, 76%) were quantitative non-randomised studies defined as any quantitative studies estimating the effectiveness of an intervention or studying other exposures that do not use randomisation to allocate units to comparison groups [29]. Of these studies, the majority involved participants that were representative of the target population (91%); used appropriate measurements regarding both the outcome and intervention (96%); and involved interventions that were administered as intended (91%). However, only 70% provided complete outcome data (with very few measuring duration or level of participation in the intervention), and only 52% accounted for confounders in the design and analysis. Of the remaining studies, three were qualitative, five were quantitative randomised controlled trials and 2 were mixed-methods. Whilst RCTs are the gold standard of intervention evaluation, those captured in this review were typically of poor quality (meeting a maximum of three criteria), with inadequate reporting or implementation of randomisation, a lack of information of blinding of the experimenters and some issues with baseline group differences. This overview indicates that designing a high-quality study that meets all quality criteria on this topic is challenging.

### The Evidence for Positive Outcomes for Children Targeted by Food Insecurity Interventions

#### Attended Interventions

Twenty-four papers included in this review related to attended interventions, including school food assistance (breakfast and lunch provision; *n* = 13), holiday clubs (*n* = 3), interventions including nutrition education (*n* = 7) and gardening clubs (*n* = 1). A summary of these papers can be found in Table 1.

##### School Food Assistance

Four papers assessed the impact of school food assistance (as a group of interventions) on children. It was found that US school food assistance can significantly improve educational difficulties, where the relationship between household food insecurity and educational difficulties disappears for children who participate [30]. It has also been found that food-insecure girls participating in the US’s School Breakfast Program (SBP), National School Lunch Program (NSLP) or Food Stamp Program (FSP—or all three) have a reduced risk of obesity compared to non-participating food-insecure girls. However, there was no effect of participation on risk of obesity for boys [31]. Moreover, participation in US school meals alongside WIC (Women, Infants and Children) and/or SNAP (Supplemental Nutrition Assistance Program) has been associated with increased risk of obesity for food-secure children, but not for food-insecure children [32]. Lastly, evidence has been found that school-based food assistance can reduce the odds of children experiencing food insecurity among high-risk (border colonias) populations [33].

Five papers evaluated the impact of school breakfast interventions on children’s outcomes, with US studies finding that they can reduce the disparity in breakfast consumption between food-secure and food-insecure children and reduce children’s food insecurity^3^ [34, 35]. UK stakeholders also perceive benefits of universal free school breakfast, including alleviating hunger and improving health outcomes, as well as providing social, behavioural and educational benefits [25]. Whilst one paper reported that UK stakeholders have concerns about universal free school breakfast increasing obesity [25], no associations between participation in US breakfast in the classroom and obesity have been found [36]. Research has failed to find evidence of gains in academic performance in relation to breakfast in the classroom in the US [36]. However, a randomised controlled trial (RCT) of UK school breakfast provision reported that post-intervention, participating children demonstrated significantly improved concentration, skipped fewer classes and ate fruit for breakfast more when compared to control children [37•]. Post-intervention, participating primary school children had higher rates of borderline/abnormal conduct and behavioural difficulties compared to the control group, and participating secondary school children more frequently had borderline/abnormal prosocial scores than the control group. However, these findings were not supported by all analyses, and a lack of baseline assessment prevents conclusions being drawn from these findings[37•].

Four papers evaluated the effects of participating in school lunch interventions. Participation in the US NSLP has been associated with increased odds of hunger [33], a health limitation, and lower maths test scores, as well as increased odds of externalising behaviour [16]. However, when comparing outcomes of siblings, one of whom does not participate in the NSLP, there are no associations of NSLP participation with negative child outcomes, suggesting that these increased odds may be a product of familial factors not controlled for elsewhere [16]. A further paper evaluated the impact of the NSLP, whilst controlling for methodological issues, such as participants self-selecting to participate in the NSLP (which has been suggested as an explanation for the positive relationship of participation with poor health), and using comparison groups of participants who were ineligible for the intervention [17]. Using this approach suggests that the NSLP reduces incidences of poor health and obesity. Other research has also explored whether the relationship between BMI and obesity is modified by participation in the NSLP [38]. However, no significant relationship between household food insecurity and child BMI was found among NSLP participants or non-participants.

##### Holiday Clubs

Three qualitative papers were detected which evaluated summer holiday clubs that offer free food alongside other enrichment activities, such as physical activity, stories and crafts. Both staff and attendees at UK holiday breakfast clubs and other holiday clubs report nutritional (e.g. more substantial and varied breakfast, trying new foods, attenuating hunger), social (e.g. removing social isolation and providing new interactions) and financial benefits to attending these clubs [26•, 27]. Lastly, parent attendees of a US lunch in the library scheme described that the intervention allowed their children to socialise, and they valued the other enrichment opportunities this intervention provided [24].

##### Nutrition Education

Six papers reported outcomes for children who had participated in food insecurity interventions involving nutrition education, with two finding no significant effects. One such paper explored the addition of six sessions of nutrition education to the Kid’s Café Program, a free meal initiative in the US, using an RCT [22]. No significant effect was found in the intervention on children’s vegetable consumption, and intervention children had significantly higher sodium intake post-intervention than control children. However, it should be noted that the authors report that there were issues with the acceptability of nutrition education classes. Another experimental paper assessed an intervention in New Zealand which offered nutrition education, fruit and vegetable tasting and encouraged growing and cooking vegetables and other healthy meals [21]. No significant effect of intervention was found on nutrient intake, but children consumed significantly fewer highly processed snack foods post-intervention. There were increases in fruit and vegetable consumption at 6 months post-intervention, but significance could not be tested due to drop out rates.

Four papers reported positive outcomes of nutrition education food insecurity interventions. A small-scale experimental study explored the effects of the Food and Fun intervention, an eight-week curriculum of nutrition education alongside education about physical activity, tasting healthy foods, free meals and physical activity [23]. The authors report that children’s fruit and vegetable consumption, as well as their levels of physical activity, significantly increased after the intervention. A similar intervention has also been implemented among sheltered homeless children, called Cooking, Healthy Eating, Fitness and Fun (CHEFF). A qualitative study found that child attendees showed some increased willingness to try different foods, developed increased liking of new foods and intentions to change health behaviours [39]. The Brighter Bites intervention in the US also has a nutrition education component, which is delivered alongside provision of free fresh produce which is redirected from food waste [40]. Parents reported that their child’s intake of fresh produce increased after participating, with most reporting that the nutrition education component was effective. Similarly, nutrition education has been integrated into a free-school meal intervention in low SES schools in parts of Greece [41]. Here, provision of a free meal, education about a healthy diet and physical activity, as well as cooking demonstrations for parents, resulted in significant increases in consumption of multiple healthful foods, and some movement towards a Mediterranean diet pattern, which is suggested to have health benefits.

Nutrition education has also been added in to subsidy interventions that seek to tackle food poverty, such as the US Supplemental Nutrition Assistance Program (SNAP-Ed). One paper evaluated the long-term effects of SNAP-Ed participation on children’s food insecurity in an RCT [42]. It was found that there was no significant change in children’s food insecurity status in comparison to a control group who received SNAP benefits alone. The authors highlight that it is likely that children are buffered from the main effects of household food insecurity (which was lowered by the intervention) and that children’s food insecurity was low in the sample at baseline.

##### Community Gardening

One paper explored the utility of a community gardening intervention for increasing vegetable intake and reducing food insecurity among child attendees. After participation, the number of children consuming vegetables several times a day increased substantially, but there was no significant change in the number of meals children missed [20].

#### Subsidy Interventions

Twenty-three papers identified in this review explored the impact of subsidy interventions on children’s outcomes. These included papers evaluating the Women, Infants and Children intervention (*n* = 4), SNAP (previously known as the Food Stamp Program; FSP) (*n* = 14), and other subsidy interventions (*n* = 6). A summary of these papers can be found in Table 2.

##### Women, Infants and Children

WIC is a short-term multi-faceted US intervention that seeks to alleviate nutritional risk among low-income women, infants and children in order to protect their health. It does this by providing nutritious foods, information on healthy eating and referrals to additional healthcare (including immunisation and screening) [43]. Four papers evaluated the possible effects of WIC on children, with the first suggesting that WIC reduces the prevalence of child food insecurity [44]. Further research has found that among WIC eligible infants, claimers had a significantly lower probability of being underweight, short or being rated as having poorer health than infants not claimed for due to access issues [45]. This, combined with the fact that infants of WIC claimants were of comparable weight and length to national averages, suggests that WIC may well attenuate nutritional and growth deficits among participants. Indeed, a further study found that WIC participation attenuates child health risks associated with family stressors, with WIC participants having higher odds of well child status and lower odds of poorer health status and overweight compared to eligible non-participants [46]. Lastly, WIC participation (along with SNAP and free school meals) has been associated with increased BMI and waist circumference for food-secure but not food-insecure children [32].

##### Supplemental Nutrition Assistance Program

SNAP is a US intervention that provides food-purchasing assistance to low-income families and individuals [43]. The outcomes of SNAP participation for children were assessed in 14 papers included in this review, making it the most extensively evaluated intervention captured. In terms of educational attainment, participation has been found to improve girls’ mathematics and reading scores [47] and moderate the negative relationship between deprivation and children’s grade attainment [48]. In terms of health outcomes, the evidence suggests that participation reduces poor health among food-insecure children [7, 18].

Evidence as to whether SNAP participation affects weight status or decreases children’s food insecurity is mixed. For example, when operating under the name the “Food Stamp Program” (FSP), participation over a five-year period was associated with decreased odds of overweight among young boys and increased odds among young girls, with no associations found for older children [49]. However, the authors acknowledge that food insecurity was not controlled for in these analyses. In other research, FSP participation has been associated with a reduced risk of overweight among food-insecure girls compared to non-participating food-insecure girls, although no significant effects were found for boys [31], and SNAP participation (along with WIC and free school meals) has been associated with increased BMI and waist circumference for food-secure but not food-insecure children [32]. Moreover, whilst one study found that, when controlling for financial stress, participation decreases both weight status and severity of overweight among children [50], a further study (which did not control for financial stress) found no relationship between food insecurity and BMI among SNAP users or non-users [38].

Findings on the effects of SNAP participation on children’s food insecurity are also mixed. Some studies have suggested that participation in SNAP is associated with decreased odds of child food insecurity among a general US population and border colonias [18, 51, 52], as well as the proportion of children not eating enough [53]. However, one study reports that there is no relationship between SNAP participation and children’s food insecurity [54] whilst a second reports that although SNAP participation reduces household food insecurity, it increases food insecurity among children [55].

##### Other Subsidy Interventions

A similar intervention to WIC is the Keeping Infants Nourished and Developing (KIND) intervention. KIND is a collaboration between primary care physicians and a food bank, providing supplementary infant formula, educational materials, and clinic and community resources. KIND intervention infants were more likely to complete a full set of well-infant healthcare visits than non-users, but there was no significant difference in weight-for-length between users and non-users [56], suggesting possible attenuation of nutritional deficits.

Two papers included in this review evaluated the Summer Electronic Benefit Transfer for Children (SEBTC), which is an extension of the standard SNAP provision. This intervention provides eligible families with an additional $60 per eligible child each month during the summer, when demands on family finances are greater. These papers reported significantly lower levels of food insecurity among randomly allocated participants, compared to a control group, as well as moderate improvements in children’s fruit, vegetable and dairy consumption [57, 58].

The Child and Adult Care Food Program (CACFP) is an American intervention that reimburses child care providers for meals and snacks consumed. One study in this review explored relationships between CACFP participation and overweight status, food consumption and food insecurity [59]. It was found that participation may reduce the prevalence of overweight among low-income children (although the authors highlight the detected effect was too small to be of note) and moderately increases consumption of vegetables and milk.

Another subsidy intervention detected in this review is the Individual Development Account (IDA) savings programme, which matches every USD saved with further two. It also provides financial education and training on budgeting and credit repair and other asset-specific training. The research presented in this review found no significant difference in children’s food insecurity between those newly enrolled on the programme and those who had graduated from the programme [60].

The final subsidy intervention detected in this review was a community-supported agriculture intervention, where low-income families can purchase a share of a farmer’s harvest at a 50% discount and then receive deliveries of fresh fruit and vegetables throughout the season. Whilst participating children had higher fruit and vegetable intake than the national average and were more likely to meet recommendations for consumption, there was no significant difference in consumption between the summer and winter when they were not receiving fresh produce [61]. Therefore, the study does not evidence a positive effect of the intervention on consumption, instead it is likely that those who chose to participate had higher consumption of fruit and vegetable consumption.

## Discussion

This review has examined the existing evidence base pertaining to interventions that have attempted to address children’s food insecurity. The review has highlighted that the existing evidence base about what works in terms tackling children’s food insecurity and the resultant potential for delivering positive impacts for children is problematic due to issues including the following: interventions having ill-defined aims; a lack of robust evaluation approaches; a lack of consistency in measures (both in terms of food insecurity and intervention outcomes); measurement of a restricted outcome or outcomes; and a lack of explanatory value. There is also considerable variation in the methods utilised to evidence the effectiveness of the interventions as some claim a broad range of positive outcomes which are measured qualitatively (e.g. self-reports) whilst other interventions are evaluated using very narrow criteria (e.g. vegetable consumption, number of healthcare visits) rather than examining the broader impact on the family in the long term. This supports concerns raised in other literature, where concepts like food insecurity are used too narrowly, with too strong a focus on outcomes of food quantity or nutrient intake [12••]. There is a lack of robust evidence of outcomes, such as that derived from RCTs. However, as outlined elsewhere, conducting such research has numerous methodological issues, particularly when related to public health interventions where implementation is rapid [37•, 62].

Several of the papers reported in this review seek to evaluate the impact of interventions on children’s food insecurity. However, food insecurity is a multi-faceted construct, with implications for food quality, variety and quantity [1]. Furthermore, there is no one internationally agreed measure of food insecurity. For example, the US government routinely collect data on food insecurity using the United States Department for Agriculture Food Security Scale, with possible outcomes ranging from high to marginal, low and very low food security. Meanwhile, the UK government does not collect such data or have an agreed standardised measure in place. Consistent measurement of food insecurity is needed not only to assess the scale and nature of the issue but also to allow robust development of interventions to address food insecurity.

Additionally, there is considerable variation in the design of interventions, with some designed to alleviate food insecurity, whilst others are designed to tackle issues that are (assumed) consequences of food insecurity and food scarcity (e.g. increasing consumption of fruit and vegetables, developing food confidence, providing nutritional education). Moreover, whilst attended interventions, such as holiday clubs, are increasingly prevalent, this review has found that they can be poorly described with very limited evaluation, and there is a paucity of comprehensive data on how, where and with whom these interventions are implemented [12••]. Typically, the interventions represented in this review lack a clear theory of change which outlines how and why the intervention might deliver the intended outcomes [63, 64]. It is therefore recommended that firstly, future research seeks to more demonstrate that interventions impact food insecurity. Secondly, plans for interventions must outline how and why the intervention will alleviate food insecurity, and therefore achieve the resultant impacts. Having done this, it will then be possible to identify how reduced food insecurity impacts on delivering particular positive outcomes for children.

The review has also revealed that there are two main strategies that have been adopted in attempts to address children’s food insecurity, which are described here as attended and subsidy interventions. These two strategies provide different possibilities for supporting families experiencing food insecurity which are also important to note. Subsidy programmes provide families with more flexibility to make decisions about how the additional resources they are provided with can best be utilised within individual families, but they have the disadvantage of potentially further stigmatising families who are defined by their low socio-economic status [65]. In contrast, the attended programmes can be devised in ways where children access support in spaces and places that they already attend (e.g. school) as universal provision which reduces the risk of children being further stigmatised [26•]. Whilst it appears that both these strategies may result in positive outcomes, it is not clear the extent to which families experiencing food insecurity are influencing the design of the interventions that they are the beneficiaries of. The disadvantage of this approach is that families do not have the opportunity to ensure that interventions best meet their complex needs in a holistic manner. Another recommendation arising from this review is therefore that systems-based approaches to tackling food insecurity are needed if real change is to be both delivered and evidenced in the long term.

## Conclusions

In summary, this review has synthesised the research that evaluates interventions to tackle children’s food insecurity and found this evidence base to be both mixed and lacking in robustness. In order to promote effective interventions to tackle children’s food insecurity, interventions should be grounded in theory of change and take a systems-based approach to both implementation and evaluation of these interventions. To do this, measurement of food insecurity must be standardised and universally implemented, ensuring that such interventions are meeting their primary aim as well as the broad variety of other positive outcomes such interventions have the potential to achieve.

## Footnotes

1 It is noted that children can move in and out of these binary groupings and may do so several times across their childhood.

2 Some papers are listed in both Tables 1 and 2 as they evaluate both attended and subsidy interventions.

3 This paper reports outcomes in terms of changes in low and very low food security as measured by the USDA Food Security Scales. For consistency and ease of interpretation, findings that have been anchored in relation to changes in food security have been used throughout this review.
